# Dietary Quality and Nutrient Intakes of Elite Paracyclists

**DOI:** 10.3390/nu16162712

**Published:** 2024-08-15

**Authors:** Keely A. Shaw, Philip D. Chilibeck, Thomas D. Warkentin, Gordon A. Zello

**Affiliations:** 1 College of Kinesiology, University of Saskatchewan, Saskatoon, SK S7N 5B5, Canada; keely.shaw@usask.ca (K.A.S.); phil.chilibeck@usask.ca (P.D.C.); 2 Crop Development Centre, Department of Plant Sciences, University of Saskatchewan, 51 Campus Dr, Saskatoon, SK S7N 5A8, Canada; tom.warkentin@usask.ca; 3 College of Pharmacy and Nutrition, University of Saskatchewan, Saskatoon, SK S7N 5B2, Canada

**Keywords:** dietary reference intake, paralympic, fibre, cycling, dietary supplements

## Abstract

Nutrient requirements for para-athletes will be influenced by a variety of factors secondary to their impairment and, therefore, recommendations for para-athletes cannot be drawn directly from that of able-bodied athletes. Information on the dietary intakes of para-athletes is lacking and therefore needs to be examined. This study assessed the nutrient intakes and diet quality of 31 paracyclists via food frequency questionnaires. Based on the dietary reference intakes, most paracyclists consumed intakes above the recommended dietary allowance (RDA) or adequate intake (AI). Recommendations were not met for iodine (males = 87% RDA; females = 62% RDA) or fibre in males (84% AI). A 26% risk of inadequacy was noted for vitamins D and E in females. A total of 42% of females and 75% of males did not meet fibre recommendations (14 g/1000 kcal), and only three athletes (all females) consumed fatty acids in the recommended omega-6 to omega-3 ratio of 4:1 or less. Athletes consumed grains, fruits, and vegetables frequently, though whole grains, pulses, and oily fish were generally consumed less often. Paracyclists appear to be consuming intakes at or above recommendations for most nutrients, though several nutrients were consumed below the recommended amounts; therefore, increasing the variety of foods consumed is suggested.

## 1. Introduction

In recent decades, parasports have experienced a substantial increase in participation. Despite this, sport science has lagged, limiting the opportunity for evidence-based practice in both health and performance for these athletes. While the evidence informing optimal nutritional practices for able-bodied individuals is abundant [1,2], the same does not exist for athletes with physical or visual impairments (i.e., para-athletes). Alterations in the physiological and metabolic functioning of para-athletes compared to their able-bodied peers make research on able-bodied athletes not directly applicable to those with physical impairments [3,4]. Physiological and structural alterations such as decreased muscle mass, spasticity, hypertonia, impaired thermoregulation, and differences in energy expenditure impact the nutritional requirements to optimize health and performance [5].

Current evidence in the area of sport nutrition for athletes with physical impairments is lacking and often restricted to wheelchair athletes [4,6]. Of the research available on other types of impairments, evidence is often limited to clinical settings, not always applying to sports performance [7,8,9]. Many paralympic sports, such as paracycling, include a variety of impairments, with both ambulatory and non-ambulatory individuals being represented [10]. Due to the variety of impairments that are represented in the sport, demands and requirements are likely to differ substantially between classes. For example, upper-body exercise (i.e., handcycling) requires less energy expenditure relative to lower-body cycling (i.e., cycling and tricycling) despite similar rates of glycogen depletion [11]. Furthermore, those with minimal physical impairment (i.e., visually impaired cyclists, C5 cyclists) are likely to have requirements similar to their able-bodied peers [10], while those with reduced muscle mass (i.e., those with spinal cord injuries or transfemoral amputations) have a reduced glycogen storage capacity, potentially impacting carbohydrate requirements before and during prolonged exercise [5]. Due to the lack of para-specific guidelines, athletes and practitioners often turn to guidelines derived from able-bodied individuals or the dietary reference intakes (DRIs) to inform decision making [5].

Current research in this area has attempted to characterize habitual intake in para-athletes, generally finding consumptions greater than the recommendations for most nutrients, though inadequate intakes are often reported for vitamin D, folate, fibre, and iron [6,12,13,14]. Of these commonly reported inadequacies, folate may be particularly important for those with brain and spinal cord injuries, as folic acid has been demonstrated to improve functional recovery and neurodevelopment [15], while vitamin D intake may be particularly vital for those with spinal cord injuries and amputations due to limitations in weight-bearing activities and implications for bone health [5]. Though some research has attempted to quantify the habitual intake in this population, no study to date has outlined the types of foods consumed by athletes in order to characterize dietary choices and consequently, how intakes may be improved. Thus, identifying potential inadequacies in different classifications of para-athletes and the foods most commonly consumed is important, not only for sport performance but also for health. Much of the above-cited research is limited by studying athletes from a variety of different sports within the same sample, potentially causing difficulties in drawing conclusions, as requirements may vary greatly across sports. Paracycling is an interesting population of athletes to study, as a variety of impairment types can be studied while controlling for the demands of the sport.

Our previous research outlined how the COVID-19 pandemic impacted the diet of elite paracyclists [16]. Given the paucity of evidence surrounding the habitual dietary intake of athletes with physical impairments, we opted to analyze the data from before the pandemic with additional participants to assess the typical intake of this population, both in terms of the types of foods consumed and habitual intake of nutrients. Therefore, the primary objective of the current research was to examine the types of foods consumed and the energy, macronutrient, and micronutrient intake provided by those foods in a group of elite paracyclists using the DRIs.

## 2. Materials and Methods

Paracyclists from English-speaking countries who had competed at the national or international level in the previous 12 months were invited through email or direct message on social media (i.e., Facebook, Instagram, or Twitter) to participate in the research. If athletes indicated their interest, a website link was provided, which took them to a food frequency questionnaire (FFQ) administered using the online platform SurveyMonkey (San Mateo, CA, USA). The first page of this questionnaire hosted the consent form for the study. Consent was implied by the participants clicking “continue” to progress to the questions pertaining to their diet. Athletes who were injured or not actively training for any reason were excluded from the research. Ethical approval was obtained from the University of Saskatchewan Research Ethics Board (BIO 3312).

### 2.1. Data Collection

Dietary intake was assessed using the Canadian Diet History Questionnaire II CDHQII [17]. This is a 165-item questionnaire that collects dietary history from the past month including food, beverages, and dietary supplements. Participants were presented with food items and asked how often they consumed that particular item in the past month (never, once per month, 2–3 times per month, once per week, 2–3 times per week, 4–5 times per week, once per day, 2+ times per day) as well as how much was consumed each time. This questionnaire was chosen because it is valid for assessing dietary intake with no differences being observed between it and a 24 h food recall [18]. Our research team has used this FFQ in past studies to capture habitual intake as well as assess diet choices [19,20]. Data from the FFQ were analyzed using the Food Processor Software (ESHA Research, Version 11.1, Salem, Orlando, FL, USA), which uses Canadian nutrient information for a given food and provides an estimate of energy, macronutrients, and micronutrients. Participants were also asked to report any other foods (type of food, frequency, and usual amount) they regularly consumed that were not asked about in the FFQ. If a food was not found in the ESHA database, nutritional information found in the governmental food database from the athlete’s home country was used.

Characterization of dietary intake was assessed by quantifying the frequency of consumption of key food groups and subcategories within those groups. Specifically, we looked at how many times per day participants consumed the following foods: fruits (fresh, frozen, dried, and canned); vegetables; grain products (bagels, bread, rice, and pasta); meat alternatives (tofu, soy meat substitutes, pulse-based soups, baked beans, and cooked dried beans); nuts and seeds; eggs (white-only and whole); meat and fish; and dairy and dairy alternatives (yogurt, cheese, and plant-based milk alternatives). We also considered the proportion of these intakes that came from whole grain sources (for grains), processed meat and fish, lean unprocessed meat, oily fish (i.e., fresh tuna, salmon, and mackerel), low- or non-fat dairy, high-protein milk alternatives (soy and pea) and low-protein plant-based milk alternatives (nut milks, rice milk, etc.).

### 2.2. Statistics

Data were assessed using JASP statistical software, version 0.10.2 (2013–2019, University of Amsterdam, Amsterdam, The Netherlands). Normality was assessed using the Shapiro–Wilks test. Due to a significant Shapiro–Wilks test, sex differences were assessed using the Mann–Whitney U non-parametric test for alcohol, protein, fibre, iodine, omega-3 fatty acids, omega-6 fatty acids, vitamins B6, B12, C, D, E, and K, folate, selenium, and caffeine. Sex differences for all other nutrients were assessed using an independent samples t-tests. Dietary intake was assessed by comparing reported intakes to the DRIs [21]. The risk of inadequacy for nutrients with an estimated average requirement (EAR) was assessed using the EAR cut-point method [13,22]. For iron, as requirements in women of reproductive age are not normally distributed, the probability approach was used [22]. In situations where a nutrient does not have a recommended dietary allowance (RDA), intakes were compared to the adequate intake (AI). If the median of the group was above the AI, it was assumed that the group’s usual intake was adequate. Intakes of nutrients for males and females as a group were also compared to the RDA by dividing the absolute intake by the RDA for their respective age group and sex, then multiplying by 100 to get a percentage intake relative to the RDA. For macronutrients, if intakes fell within the acceptable macronutrient distribution ranges (carbohydrate: 45–65%, protein: 10–35%, fat: 20–35% of daily caloric intake), intake was assumed to be sufficient for health. Individual data for nutrients with a tolerable upper intake level (UL) (vitamin A, vitamin C, vitamin D, vitamin E, vitamin B_3_, vitamin B_6_, folate, calcium, iodine, iron, magnesium, phosphorus, selenium, and zinc) were assessed to gauge the risk of toxicity. Differences in dietary intake with and without supplementation were assessed using a dependent samples *t*-test. Differences in intakes between sport classes (C, H, T, and B) were characterized to enhance the generalizability of results, though due to the limited sample size in some classes, statistical comparisons were not made. Significance was accepted at *p* < 0.05. All data are presented as means ± standard deviation.

## 3. Results

Thirty-four individuals consented to the research and began the FFQ. Three individuals (all males) did not complete the FFQ; thus, thirty-one paracyclists were included in the analysis (n =19 females; n = 12 males; average age = 38 years; age range: 21–60 years). Twenty-eight of these individuals were classified as “elite” (i.e., race for and are supported by their national body) while three were sub-elite (i.e., competed at the national level but not on the national team). Participants were residents of the following countries: Australia (n = 8); Belgium (n = 1); Canada (n = 10); Great Britain (n = 2); Ireland (n = 1); South Africa (n = 1); and the USA (n = 7). The participant sport class classification is displayed in Table 1.

### 3.1. Dietary Choices

The daily frequency of consumption for food items of interest is presented in Table 2. Generally, grains, fruits, and vegetables were consumed frequently (3.5, 2.9, and 4.2 times per day, respectively), though the proportion of grains that came from whole sources was low (18%). Meat alternatives (pulses) and nuts and seeds were consumed less frequently (0.31 and 0.69 times per day, respectively). Dairy and dairy alternatives were consumed 1.75 times per day, with approximately half of all dairy intake coming from low- or non-fat dairy (53%), and 11% coming from low-protein milk alternatives (i.e., almond milk and rice milk).

### 3.2. Macronutrient and Energy Intake

Consumption of all macronutrients was aligned with acceptable macronutrient distribution ranges (Table 3). Males consumed significantly more total fat (*p* = 0.048) and saturated fat (*p* = 0.01) compared to females. Both males and females consumed less than 10% of their total daily energy from saturated fat, as recommended by the World Health Organization [23]. Macronutrient intake classified by sport class is displayed in Table 4. Tandem athletes consumed more energy and greater amounts of all macronutrients compared to the other classifications. As a percentage of the total intake, consumption of the different macronutrients was comparable across all classifications. The consumption of macronutrients and energy were comparable between cyclists, handcyclists, and tricyclists, though cyclists appear to have consumed more fibre (33.4 ± 16.1 g) compared to handcyclists and tricyclists (28.0 ± 6.6 g and 28.7 ± 12.2 g, respectively).

**Figure 1:**
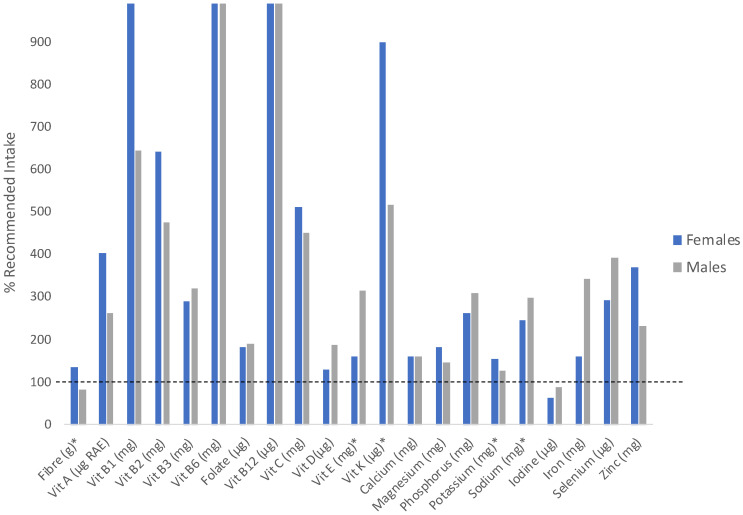
Intakes of male and female paracyclists expressed as a percent of daily recommended intake. The dashed line represents the recommended intake. Nutrients with * indicate reference values are at adequate intake. Reference values for all other nutrients are recommended dietary allowances. Female intakes of vitamin B1 and male and female intakes of vitamin B6 and B12 exceed 1000% of the recommended intake. RAE = retinol activity equivalents.

When considering the AI for fibre (38 g/day for males, 25 g/day for females), adequate fibre was consumed by females (33 g/day) but not males (29 g/day) (Figure 1). When fibre intake was assessed based on relative recommendations (i.e., relative to caloric intake; 14 g/1000 kcal), 42% of females (n = 8) and 75% of males (n = 9) did not meet recommendations [21].

Males consumed an average of 19.3 ± 7.8 g and 2.1 ± 0.9 g of omega-6 and omega-3 fatty acids, respectively, while females consumed an average of 15.7 ± 8.3 g and 2.1 ± 1.2 g of omega-6 and omega-3 fatty acids, respectively. These intakes exceed the AI values of 17 g/day and 1.6 g/day for intakes of omega-6 and omega-3 fatty acids in males and 12 g/day and 1.1 g/day for intakes of omega-6 and omega-3 fatty acids in females [21]. Notably, only three athletes (all females) consumed omega-6 to omega-3 fatty acids in a ratio of 4:1 or less as recommended [24]. The ratio of omega-6 to omega-3 was not different between the sexes (*p* = 0.51).

### 3.3. Micronutrient Intake

**Figure 2:**
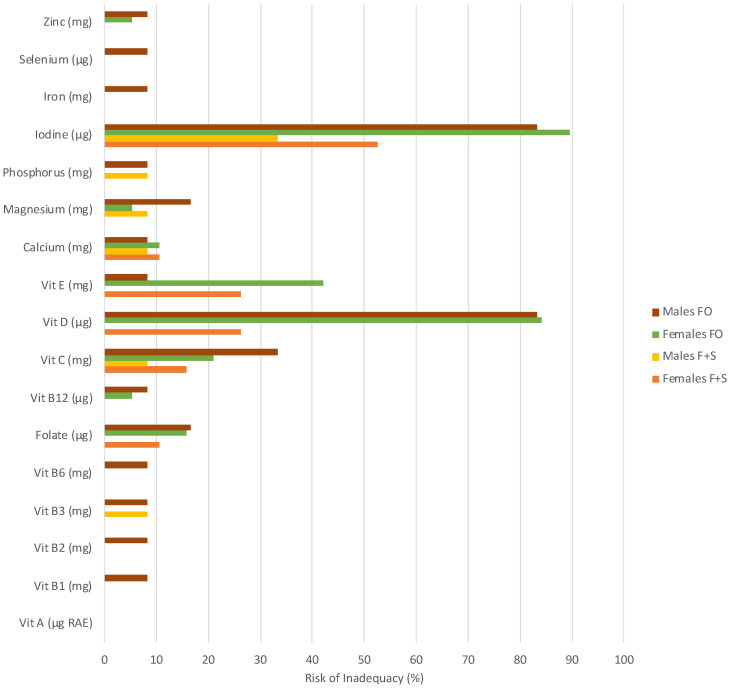
The estimated prevalence of inadequacy (%) based on EAR cut-point method for nutrients from food-only (FO) and food-plus-supplement intake (F+S) in male and female paracyclists. RAE = retinol activity equivalents. Where no bars are present, the risk of inadequacy was below determinable.

Dietary intake by sex, including supplements, is displayed in Table 3. Males consumed significantly more selenium compared to females (*p* = 0.04). No other differences were evident between the sexes. A high risk of inadequacy was observed in both males and females for iodine (males 33% risk; females 53% risk). Females exhibited a high risk of inadequacy for vitamin D (26%) and vitamin E (26%), while males did not (Figure 2). Micronutrient intake appears to be relatively similar between sport classes, with tandem athletes consuming greater amounts of most micronutrients compared to other sport classes, except for vitamins E, C, B_1_, B_2_, B_6_, and B_12_ (Table 4).

When group averages were compared to recommended intakes (RDA or AI), most micronutrients were consumed at intakes meeting or exceeding recommendations, with the exception of iodine (Figure 1). When considered as a group average, no nutrients were consumed in excess of the UL. However, when assessed individually, intakes greater than the UL were consumed by five individuals for vitamin B_3_ (one male, four females), six individuals for vitamin B_6_ (one male, five females), three individuals for calcium (all females), two individuals for iron (both females), and six individuals for zinc (one male, five females). The average consumption over the UL was minimal for calcium (1%), moderate for iron (12%), but rather substantial for vitamin B_3_ (237% above UL), B_6_ (72% above UL), and zinc (75% above UL).

### 3.4. Supplement Use

The use of supplements was reported by 27 (87%) athletes. The most commonly used supplements were vitamin D (n = 16), vitamin C (n = 14), multivitamins and mineral supplements (n = 12), probiotics (n = 10), and omega-3/fish oil (n = 10). Other reported supplements included iron (n = 8), magnesium (n = 7), sport supplements (n = 6) (our questionnaire did not specify which sport supplements), calcium (n = 6), zinc (n = 6), vitamin B complex (n = 5), glucosamine (n = 5), vitamin B12 (n = 4), coenzyme Q10 (n = 2), echinacea (n = 2), garlic (n = 2), ginger (n = 2), vitamin E (n = 1), and folic acid (n = 1). Supplement consumption significantly increased intakes for vitamin B1 (*p* = 0.01), vitamin B2 (*p* = 0.002), vitamin B12 (*p* = 0.01), vitamin C (*p* = 0.001), vitamin D (*p* < 0.001), folate (*p* = 0.005), iodine (*p* < 0.001), iron (*p* = 0.002), and zinc (*p* = 0.001). The risk of inadequacy was slightly increased (<10% increase in risk) when considering food-only intake compared to both food and supplement intake in males for vitamin B1, vitamin B2, vitamin B6, vitamin E, iron, and selenium and in females for folate and vitamin C. Both sexes had a slight increase in the risk of inadequacy (<10% increase in risk) for vitamin B12, magnesium, and zinc when supplements were not considered. A notable increase in the risk of inadequacy (>10% increase in risk) was observed for vitamin C and folate in males (25% and 17% increase in risk, respectively) and vitamin E for females (16% increase in risk). Both sexes had a notable increase in risk (>10%) for vitamin D (males: 83% increase in risk; females: 57% increase in risk) and iodine (50% increase in risk for males, 37% increase in risk for females) when considering food only (Figure 2). Of those who consumed over the UL for the above-noted nutrients, all supplemented with the nutrients they consumed in excess except one female for both vitamin B_6_ and calcium, for which intakes came from a diet-only intake.

### 3.5. Caffeine Intake

No differences were evident between males and females for caffeine intake (*p* = 0.25). Males consumed an average of 179 ± 245 mg/day, while females consumed 174 ± 99 mg/day.

### 3.6. Alcohol Intake

Thirteen individuals reported consuming no alcohol (eight females and five males). There were no differences between males and females, with males consuming an average of 1.6 ± 1.9 g of alcohol per day and females consuming 2.1 ± 3.3 g per day (*p* = 1.0). When considering only those who consumed alcohol (i.e., removing the zero data points), males consumed 2.8 ± 1.7 g per day and females consumed 3.6 ± 3.7 g per day (*p* = 0.55).

## 4. Discussion

This study aimed to characterize the dietary intakes of elite paracyclists and assess intakes against the DRIs to inform athletes and practitioners (i.e., coaches, dieticians, etc.) of possible shortcomings in the diet of paracyclists and strategies for improvement. We found that males and females largely met or exceeded the RDA for most nutrients with a few exceptions. Athletes consumed fruits, vegetables, and grains frequently, with the consumption of whole grains and meat alternatives being quite low. The intakes of fruit and vegetables consumed by athletes in this study are generally in line with the recommendations of two servings of fruit and three servings of vegetables daily, which is associated with lower mortality [25]. However, intakes of whole grain products appear to be well below recommendations from around the world [26]. The low intake of whole grains is similar to the findings of Joaquim et al. [27] in track and field paralympic athletes, though this group also observed reduced intakes of fruits, which is contrary to the current research. The current research, however, is different from that reported in able-bodied cyclists by Matusiak-Wieczorek et al. [28], who observed increased consumption of wholegrain cereal products compared to cereal products from refined grains. Notably, Matusiak-Wieczorek et al. studied amateur cyclists, who may have been more cognizant of dietary recommendations for health rather than performance-centric goals. Intakes of legumes appear to be similar in the current sample compared to the general population, as Miller et al. [29] found legume consumption to be 0.4 times per day. The intake reported in the current research (0.31 times per day) is lower than most recommendations globally, where the consumption of legumes is recommended daily [30]. However, dairy intake appears to be generally in line with global recommendations, in which most countries recommend a daily intake [31]. Of those who chose plant-based milk alternatives, very little was from high-protein milk alternatives (i.e., soy and pea milk). Soy and pea milks are generally considered to be nutritionally similar to cow’s milk due to the high protein content, (cow’s milk = 8 g/240 mL; soy milk = 7 g/240 mL), while other plant-based milk alternatives (i.e., almond and other nut milks, rice milk, and coconut milk) have 0–1 g/240 mL [32]. This may be an important consideration for athletes who are aiming to replace dairy milk with a plant-based alternative while getting the same nutritional benefits.

### 4.1. Energy and Macronutrient Intake

No statistical difference was observed between the sexes in relation to energy intake, although males had an increased intake of energy compared to their female counterparts approaching significance (*p* = 0.06). Similarly, the intakes of carbohydrates and proteins were greater in males than females, though not significantly so. Males did, however, consume significantly more total fat and saturated fat, though when expressed relative to the total caloric intake, a significant difference was no longer evident. Differences in energy intake between the sexes are expected, as well as the large range of intake in athletes with impairments [6,12,31,33]. The variability in intakes supports the need for individualized recommendations in this population. Intakes of each macronutrient as a percentage of the total caloric intake fall within the acceptable macronutrient distribution ranges, suggesting that the proportion of energy from each macronutrient is likely sufficient for the maintenance of health. However, carbohydrate intakes for both males and females were at the low end of the acceptable macronutrient distribution range (45–65% of the daily caloric intake). As athletes, in particular endurance athletes, require higher carbohydrate intakes to support training and recovery, greater carbohydrate intakes are likely required to fuel optimal performance [1]. The consumption of protein as a percentage of the total energy intake observed in this research is similar to that observed in able-bodied cyclists ([34]; 21% in the current research vs. 19%), though the distribution of fat and carbohydrates was skewed rather notably toward fat intake compared to that observed in able-bodied cyclists (fat = 31% in the current research vs. 21%; carbohydrates = 49% in the current research vs. 59%). Athletes and support staff should monitor measures such as body weight and training tolerance to assess the suitability of the total energy intake and encourage adequate carbohydrate intake to optimize health and performance.

The current research reported no differences in carbohydrate or protein intake between males and females, although males consumed more total and saturated fat than females. Our results are similar to those observed by Gerrish et al. [35], who found no statistical differences between males and females in terms of macronutrient intake in athletes with spinal cord injury, as well as no differences in energy intake. Our results differ from those observed by Goosey-Tolfrey and Crosland [12] and Madden et al. [6], who reported that females consumed lower intakes of protein and carbohydrates, respectively. Comparing the current research to those mentioned above should be performed with caution, as Gerrish et al. [35] and Goosey-Tolfrey and Crosland [12] studied wheelchair athletes, while our research included only four wheelchair athletes (i.e., handcyclists). Participants studied by Madden et al. [6] were not adequately described to determine the level of impairments. The majority of respondents in the current research were ambulatory (i.e., cyclists), making direct comparisons with previous research difficult.

The results of this research are particularly unique, as we have characterized the omega-3 and -6 intakes of the athletes. While much research has assessed the health impacts of omega-3 fatty acids [36], looking at both of these fatty acids together may provide more insight into potential health implications rather than looking at either omega-3 or omega-6 fatty acids independently. Reducing the omega-6 to omega-3 ratio in the diet reduces cardiovascular disease, inflammation, and overall mortality, with ratios of 4:1 (omega-6/omega-3) or less being considered optimal [24]. Ratios of 15:1 or 20:1 are frequently described in a typical Western diet [24]. Males and females in our sample had ratios of 8.7:1 and 8.0:1, respectively. While this is a marked improvement compared to findings in the general population, further improvements in health may be gained by reducing this ratio. Our research found intakes of fatty fish to be 0.16 times per day, a frequency which aligns with approximately one time per week. Most countries recommend eating fatty fish at least twice a week [37]. A para-athlete population in particular may benefit from including more sources of omega-3 fatty acids in their diet, such as fatty fish, walnuts, flaxseeds, and soybeans to improve this ratio, which may reduce inflammation and oxidative stress, states which are common in those with chronic conditions [4].

The insufficient intake of fibre observed in the current research (42% of females and 75% of males consuming less than 14 g/1000 kcal) is in line with the findings of Eskici and Ersoy [38] (wheelchair basketball) as well as Krempien and Barr [13] (athletes with spinal cord injury), though contrary to the findings of Duarte et al. [39] (para-athletes). Differences between the current research and the findings of Duarte et al. [39] are likely due to the athletes in the current research consuming notably less carbohydrates than in the sample described by Duarte (272 g/day vs. 512 g/day). While athletes may opt for foods lower in fibre to decrease feelings of satiety and meet their energy needs [1], sufficient intakes of fibre are still important to regulate fecal bulk [21,40] and to limit the risk of hypercholesterolemia and metabolic conditions [41]. Decreasing the risk for hypercholesteremia and metabolic conditions may be important for this population given the increased age of many para-athletes and the potentially decreased muscle activation during regular activities in non-ambulatory athletes [42]. This may also be important for athletes with spinal cord injuries to reduce instances of constipation [40,41], although excessive intake of fibre may exacerbate symptoms and thus intakes should be adjusted as necessary to achieve desired bowel function [43,44]. Intakes between 18–31 g per day of fibre have been recommended for those with spinal cord injury [45], which is lower than the current DRIs for males (38 g/day) but in line with DRIs for females (25 g/day). As fibre acts as a prebiotic, favorably impacting the gut microbiota [46], it has the potential to enact downstream effects on different systems by way of the gut microbiome. Improvements in gut microbiota may also assist with gastrointestinal concerns that may be experienced by elite athletes who travel frequently around the world for competition. Though the direct impact of the microbiome on exercise performance remains in its infancy, the recent literature found reductions in the perception of effort during exercise in young basketball players despite no increase in performance following supplementation with soluble fibre [47].

The current research found consumption of whole grain products, pulses (e.g., lentils, chickpeas, beans, and yellow peas), and nuts and seeds to be quite low (approximately four, two, and four times per week, respectively). Increasing the diversity of the diet to include more food sources from these food groups may assist in meeting fibre recommendations and supporting a healthy gut microbiome.

### 4.2. Micronutrients

Given the lack of specific recommendations for an athletic population or for those with physical impairments, the intakes of the current sample have been assessed compared to the DRIs [22]. Our results are similar to much of the literature, observing intakes meeting or exceeding recommendations in this population for most micronutrients [6,12,14,31,43,48]. Though recommendations for specific micronutrient intakes in para-athletes are not known, exceeding the DRI values is probably beneficial as the stressing of metabolic pathways induced by exercise may increase the requirements for some micronutrients [1]. No nutrient was consumed in excess of the UL at the group level, though some individuals consumed more than the UL for vitamin B_3_, vitamin B_6_, calcium, iron, and zinc. Of those who consumed more than the UL, most excess intakes were achieved through supplementation. Consuming nutrient intakes above the UL has no known benefit and increases the risk for adverse effects [49]. Athletes should be aware of their general nutrient intake so as to not exceed the UL for specific nutrients, and only supplement when necessary, as advised by nutrition professionals.

The results of the current study differ from much of the published literature, which has found low intakes of vitamin D in a para-athlete population [12,13,14,27,45,50]. In our current sample of elite paracyclists, the risk of inadequacy for vitamin D intake was quite high when considering levels from food-only intake but was largely corrected through supplementation. Some of this research [13,14,45,50] specified that supplements were considered in their analysis, though it is unclear whether supplements were considered in the reporting of dietary intake by Madden et al. [6] and Goosey-Tolfrey and Crosland [12]. Our results also differ from much of the literature concerning intakes of calcium and magnesium, as Krempien and Barr [13] and Sasaki and Da Costa [14] have reported inadequate intakes relative to guidelines. Calcium and magnesium may be especially important for athletes with physical disabilities, as deficiencies may lead to a decline in neuromuscular function and impair sport performance [51]. Differences across studies could be related to the method of collection (food records vs. FFQ), the sports studied, or the population studied (ambulatory vs. non-ambulatory athletes; type of impairment).

Although the majority of nutrients were consumed at or above recommendations, intakes of iodine were below recommendations in both males and females. Iodine is an essential component of thyroid hormones, which are particularly important for myelination in the central nervous system [22]. Optimizing iodine may therefore be of particular interest in para-athletes with conditions in which myelination is compromised, such as multiple sclerosis and Charcot–Marie–Tooth disease, as insufficient intakes may lead to an acceleration in functional degeneration. For any athlete, able-bodied or para, iodine is important to optimize thyroid function and metabolism [52], and thus is important for maintaining the energy levels required for training and recovery. As intense or prolonged exercise may impact iodine homeostasis in the body [53], athletes with high training loads should ensure adequate consumption to allow for optimal performance and recovery from exercise. Given that iodine is consumed through iodized table salt, which was not directly measured in the current research, it is likely that intakes of iodine were underestimated. Other sources of iodine (besides iodized table salt) include seaweed, shellfish, eggs, and dairy.

### 4.3. Alcohol and Caffeine

Evidence suggests alcohol to be deleterious pre- and during training due to impacts on metabolism, thermoregulation, and skills/concentration. As such, the Academy of Nutrition and Dietetics, Dietitians of Canada, and the American College of Sports Medicine recommend athletes consider public health guidelines around alcohol consumption and consider minimizing or avoiding alcohol consumption [1]. While public health policies vary from country to country, the World Health Organization reports that alcohol is responsible for three million deaths annually, with no safe consumption level [54]. In Canada, public health now recommends reducing alcohol intake, regardless of your current habits, and limiting intake to two standard drinks per week to minimize health risks [55]. The athletes in the current sample reported consuming ~2 g of alcohol per day (3 g per day when not including those who abstain), which is equivalent to approximately one to 1.5 drinks per week.

Caffeine intake has been studied widely in an athletic context for its ergogenic effect on a variety of types of exercise performance [56,57,58]. Health Canada suggests 400 mg daily to be an amount well-tolerated by most individuals [59]. Caffeine intake in the current sample was reported at 176 mg per day, well below the recommended upper limit. The current questionnaire, however, did not take into account race day habits, during which caffeine intake may increase substantially.

### 4.4. Strengths and Limitations

Our study is strengthened by studying a single subgroup of para-athletes (i.e., paracyclists), therefore controlling for the demands of the sport. However, by choosing to include a single subset of para-athletes, we are limited in the population from which we can draw, limiting our sample size. That said, the most recent Para-cycling Road World Championships (Glasgow, 2023) had 427 athletes registered to compete. Our sample size is 7.3% of this registration number, indicating our sample is not an insignificant proportion of the total population. Regardless, the limited sample size may impact our ability to find statistically significant differences in the data. The inclusion of only English-speaking countries may also limit the generalizability of the results due to cultural biases. Future research should consider comparing paracyclists to a broader cohort of para-athletes to better contextualize the findings and should consider the socioeconomic background of the participants studied, as research has found that characteristics such as education status and income may impact the diversity and quality of dietary intake [60,61]. Our study assessed the intake of specific polyunsaturated fatty acids, which is a novel finding unique to our research. However, our study is limited by the distribution of athletes from the different sport classes (C, H, T, and B). Of the 31 participants included, over half were cyclists (n = 17), with significantly fewer tandem riders (n = 3), handcyclists (n = 4), and tricyclists (n = 7). Therefore, making comparisons between classes (and different severities/types of impairment) is difficult. The FFQ used relies on recalling the consumption of various foods over a period of time, which could result in recall error and does not include strategies to ensure accuracy of reporting. However, Labonté et al. [62] have found this questionnaire to be both valid and reproducible at both the individual and group levels. Furthermore, we analyzed FFQ responses based on Canadian nutritional information and guidelines, which may vary slightly in other countries. We also did not collect body weight data of the participants in this study, so we cannot characterize macronutrient intake relative to body mass, which is how current sport nutrition guidelines are reported. Even with these limitations, any dietary information in this population is novel.

## 5. Conclusions

The current research has provided information on the dietary intake and food choices of elite paracyclists, which will be useful to coaches and healthcare providers working with para-athletes to help them optimize their nutrition to maximize health and performance. Specifically, athletes should be encouraged to consume adequate carbohydrates to support endurance exercise and to focus on consuming a variety of minimally processed, whole food options such as whole grains, pulses, and legumes to improve dietary quality. By focusing on a variety of whole food products, athletes can ensure they consume adequate intakes of macronutrients and micronutrients without the need to supplement, enhancing both health and performance outcomes. Furthermore, for those who do not consume dairy milk, a high-protein alternative (i.e., soy or pea) should be considered rather than low-protein options such as rice or almond. Future research should investigate how intakes differ between different types and degrees of impairment and work toward generating dietary recommendations for this population.

## Figures and Tables

**Table 1:** Sport classification of participants (n = 31).

Bike Class	Males (n = 12)	Females (n = 19)
B	2	1
C2	1	0
C3	1	3
C4	1	8
C5	2	1
T1	0	1
T2	2	4
H1	1	0
H2	1	0
H3	1	0
H4	0	1

Coding of classification is based on sport classification system; H = handbike; T = tricycle; C = bicycle; B = tandem. Lower numbers (ex., C1) indicate a greater degree of impairment.

**Table 2:** Daily intake of food groups and types.

Food Group	Frequency (Per Day)		
	Males (n = 12)	Females (n = 19)	Overall (n = 31)
Grains	3.19	3.75	3.5
Of which are whole grains	0.57	0.70	0.63
Meat Alternatives	0.20	0.39	0.31
Vegetables	3.81	4.48	4.20
Fruit	2.77	2.91	2.85
Nuts and Seeds	0.71	0.71	0.71
Meat and fish	230	2.08	2.12
Of which is lean unprocessed *	1.18	0.85	0.98
Of which is processed	0.32	0.31	0.31
Of which is oily fish	0.21	0.10	0.16
Eggs	0.65	0.49	0.54
Dairy and Dairy Alternatives	1.31	1.04	1.20
Of which are low- or non-fat dairy	1.00	0.83	0.92
Of which is soy milk	0.01	0.01	0.01
Of which are other plant-based dairy alternatives	0.12	0.21	0.20

* does not include fish; meat alternatives include tofu and other soy products, dried and cooked pulses (lentils, beans, and peas), and baked beans; plant-based dairy alternatives include almond and rice milk alternatives.

**Table 3:** Daily dietary intake of paracyclists by sex.

Nutrient	Males (n = 12)	Females (n = 19)
Energy (kcal)	2563 ± 946	2015 ± 631
Carbohydrate (g)	308 ± 116	248 ± 87
Carbohydrate (% kcal)	48.0 ± 2	50.0 ± 6.5
Total fibre (g)	29.5 ± 9.8	33.1 ± 16.1
Protein (g)	132 ± 59	109 ± 51
Protein (% kcal)	20.1 ± 4.3	22.1 ± 5.6
Total fat intake (g)	90 ± 36	68 ± 25
Total fat intake (% kcal)	32.4 ± 9.0	28.9 ± 3.7
Saturated fat (g)	26 ± 12	17 ± 6
Saturated fat (% kcal)	8.4 ± 2.1	7.9 ± 1.8
Monounsaturated fat (g)	32 ± 13	24 ± 12
Polyunsaturated fat (g)	23 ± 9	19 ± 9
Omega-3 fatty acid (g)	2.1 ± 0.9	2.1 ± 1.2
Omega-6 fatty acid (g)	19.3 ± 7.8	15.7 ± 8.3
Omega-6/omega-3 ratio	8.7 ± 3.0	8.0 ± 2.8
Vitamin A (µg RAE)	2132 ± 519	2818 ± 2262
Vitamin B1 (mg)	7.7 ± 14.0	13.4 ± 21.7
Vitamin B2 (mg)	6.1 ± 3.8	7.1 ± 7.2
Vitamin B3 (mg)	51.5 ± 22.0	40.8 ± 17.8
Vitamin B6 (mg)	18.4 ± 43.4	51.0 ± 79.7
Folate (µg)	726 ± 209	727 ± 405
Vitamin B12 (µg)	96.0 ± 286.6	272.8 ± 469.4
Vitamin C (mg)	333 ± 430	383 ± 430
Vitamin D (µg)	28.3 ± 14.9	19.6 ± 12.2
Vitamin E (mg)	46 ± 74	24 ± 16
Vitamin K (µg)	513 ± 279	811 ± 814
Calcium (mg)	1558 ± 474	1590 ± 608
Magnesium (mg)	589 ± 138	583 ± 209
Phosphorus (mg)	2070 ± 798	1830 ± 732
Potassium (mg)	4033 ± 1632	3980 ± 1480
Sodium (mg)	4410 ± 1511	3635 ± 1348
Iodine (µg)	130 ± 60	93 ± 71
Iron (mg)	26.8 ± 8.0	29.1 ± 11.9
Selenium (µg)	213 ± 70	161 ± 67
Zinc (mg)	25.6 ± 17.7	29.6 ± 24.9

Intakes include consumption of both food and supplementation; RAE = retinol activity equivalent.

**Table 4:** Daily dietary intake of paracyclists by sport class.

Nutrient	Handbike (n = 4)	Bicycle (n = 17)	Tandem (n = 3)	Tricycle (n = 7)
Energy (kcal)	2143 ± 694	2185 ± 809	3003 ± 384	2063 ± 922
Carbohydrates (g)	242 ± 68	273 ± 116	333 ± 34	260 ± 103
Carbohydrate (% kcal)	45.6 ± 5.5	49.4 ± 7.9	44.5 ± 2.7	52.8 ± 8.3
Total Fibre (g)	28.0 ± 6.6	33.4 ± 16.1	43.1 ± 15.9	28.7 ± 12.2
Protein (g)	114 ± 50	117 ± 50	191 ± 81	103 ± 51
Protein (% kcal)	20.8 ± 3.2	21.8 ± 5.7	24.9 ± 7.7	19.1 ± 3.2
Total Fat (g)	81 ± 27	73 ± 31	106 ± 11	68 ± 36
Total Fat (% kcal)	34.1 ± 3.4	30.1 ± 7.5	32.4 ± 6.9	28.3 ± 5.6
Saturated fat (g)	22 ± 8	19 ± 9	27 ± 4	20 ± 13
Saturated fat (% kcal)	9.1 ± 1.3	7.9 ± 2.0	8.1 ± 1.7	8.2 ± 2.2
Monounsaturated fat (g)	29 ± 12	26 ± 13	39 ± 7	23 ± 12
Polyunsaturated fat (g)	22 ± 6	20 ± 10	29 ± 5	17 ± 8
Omega-3 Fatty Acid (g)	2.5 ± 0.9	2.1 ± 1.2	3.5 ± 1.5	1.8 ± 0.9
Omega-6 Fatty Acid (g)	17.5 ± 6.4	16.8 ± 9.2	23.5 ± 5.8	14.6 ± 6.7
Omega-6/omega-3 ratio	7.7 ± 3.7	8.5 ± 2.4	8.3 ± 5.9	7.9 ± 1.7
Vitamin A (µg RAE)	1669 ± 606	2840 ± 2263	3676 ± 1606	2293 ± 1315
Vitamin B1 (mg)	15.0 ± 24.6	12.1 ± 20.0	4.2 ± 1.0	9.9 ± 19.8
Vitamin B2 (mg)	7.4 ± 6.8	7.1 ± 6.2	5.9 ± 1.4	5.9 ± 7.3
Vitamin B3 (mg)	39.1 ± 19.3	44.0 ± 18.0	71.2 ± 22.0	39.3 ± 19.0
Vitamin B6 (mg)	41.3 ± 75.5	38.7 ± 74.2	9.8 ± 4.1	48.2 ± 75.8
Folate (µg)	654 ± 145	709 ± 383	984 ± 298	771 ± 399
Vitamin B12 (µg)	322.0 ± 621.9	230.9 ± 423.7	16.6 ± 3.6	153.2 ± 382.1
Vitamin C (mg)	155 ± 66	309 ± 386	517 ± 455	675 ± 539
Vitamin D (µg)	31.2 ± 7.0	18.5 ± 11.7	34.6 ± 15.1	24.0 ± 17.6
Vitamin E(mg)	23 ± 9	38 ± 64	34 ± 13	26 ± 16
Vitamin K (µg)	460 ± 175	751 ± 745	1263 ± 898	636 ± 594
Calcium (mg)	1633 ± 342	1594 ± 509	1924 ± 586	1430 ± 757
Magnesium (mg)	511 ± 150	581 ± 180	829 ± 117	564 ± 220
Phosphorus (mg)	1662 ± 535	1974 ± 694	2996 ± 916	1637 ± 797
Potassium (mg)	3571 ± 1251	4068 ± 1430	5827 ± 2184	3746 ± 1654
Sodium (mg)	3778 ± 1199	3863 ± 1483	5309 ± 1531	3695 ± 1441
Iodine (µg)	79 ± 61	108 ± 66	143 ± 91	109 ± 80
Iron (mg)	22.4 ± 2.0	31.4 ± 11.6	32.5 ± 4.9	23.2 ± 10.7
Selenium (µg)	161 ± 52	183 ± 71	265 ± 68	157 ± 74
Zinc (mg)	25.6 ± 17.7	29.6 ± 24.9		25.4 ± 25.0

Intakes include consumption of both food and supplementation; RAE = retinol activity equivalent.

## Data Availability

Data are available upon request.
